# Influence of NF_3_ Plasma-Treated HfO_2_ Gate Insulator Surface on Tin Oxide Thin-Film Transistors

**DOI:** 10.3390/ma16227172

**Published:** 2023-11-15

**Authors:** Christophe Avis, Jin Jang

**Affiliations:** Advanced Display Research Center, Department of Information Display, Kyung Hee University, Seoul 130-701, Republic of Korea; jjang@khu.ac.kr

**Keywords:** thin-film transistor, tin oxide, oxide semiconductor, NF_3_ plasma treatment, crystallinity

## Abstract

We studied the impact of NF_3_ plasma treatment on the HfO_2_ gate insulator of amorphous tin oxide (a-SnO_x_) thin-film transistors (TFTs). The plasma treatment was for 0, 10, or 30 s. The HfO_2_ insulator demonstrated a slightly higher breakdown voltage, whereas the capacitance value remained almost constant (~150 nF/cm^2^). The linear mobility slightly increased from ~30 to ~35 cm^2^/Vs when the treatment time increased from 0 to 10 s, whereas a 30 s-treated TFT demonstrated a decreased mobility of ~15 cm^2^/Vs. The subthreshold swing and the threshold voltage remained in the 100–120 mV/dec. range and near 0 V, respectively. The hysteresis dramatically decreased from ~0.5 V to 0 V when a 10 s treatment was applied, and the 10 s-treated TFT demonstrated the best stability under high current stress (HCS) of 100 μA. The analysis of the tin oxide thin film crystallinity and oxygen environment demonstrated that the a-SnO_x_ remained amorphous, whereas more metal–oxygen bonds were formed with a 10 s NF_3_ plasma treatment. We also demonstrate that the density of states (DOS) significantly decreased in the 10 s-treated TFT compared to the other conditions. The stability under HCS was attributed to the HfO_2_/a-SnO_x_ interface quality.

## 1. Introduction

Amorphous oxide semiconductors (AOSs), among which is indium gallium zinc oxide (IGZO) [1], have been widely investigated for their potential uses in large area electronics like active-matrix organic light-emitting diodes (AMOLEDs). Until recently, only multiple-cation-based AOSs have demonstrated an amorphous phase [2,3]. Nevertheless, the mono-cation-based AOS tin oxide was shown to have an amorphous phase (a-SnO_x_) by careful choice of fabrication process [4,5].

Reaching Hall mobilities of ~10 cm^2^/Vs, with bandgaps over 3 eV, the AOSs have demonstrated fundamental characteristics for potential uses in industrial applications requiring thin-film transistors (TFTs) [1,2,3]. The essential parameters of the TFTs are the mobility (μ_lin_), the threshold voltage (V_th_), the subthreshold swing (S.S.), and the stability over various types of stresses. Various channel materials have been investigated in AOS TFTs, from IGZO to zinc tin oxide (ZTO) [6]; indium zinc tin oxide (IZTO) [7,8]; and, more recently, indium gallium tin oxide (IGTO) [9,10], which demonstrated high mobilities, reaching 50 cm^2^/Vs, and also enhancements of mobilities with the use of high-k dielectrics were demonstrated [2,3,11,12]. In addition, atomic layer deposition (ALD) has demonstrated various advantages over conventional sputtering. IZTO and IGZO TFTs have demonstrated high mobilities over 10 cm^2^/Vs [13,14]. Also, due to the thickness monitoring, multiple thin layers (less than 10 nm each) are possible to achieve. IGZO TFTs with Ga_2_O_3_, In_2_O_3_, and ZnO multiple layer stacks were demonstrated and showed mobilities reaching 100 cm^2^/Vs [15].

Other than the conventional vacuum process, the solution process has been investigated and can lead to performances close to or even superior to the vacuum-processed TFTs [5,13,16,17]. Even though solution processes can compete in terms of energy budgets (i.e., annealing temperature), high-temperature solution-processed materials and devices are also of interest. Indeed, spin coating [18,19], inkjet printing [20,21], and spray pyrolysis [22,23] have demonstrated TFTs with mobilities over 10 cm^2^/Vs.

Multiple atom-based AOSs are of interest as they are amorphous, and mono-atomic oxide semiconductors, like ZnO, In_2_O_3_, or SnO_2_, are usually polycrystalline. Tin oxide has recently demonstrated high performance TFTs through various methods. From vacuum to solution processes, TFTs have demonstrated mobilities over 50 cm^2^/Vs [19,24,25,26]. We demonstrated that by combining UV treatments and high-k dielectrics, tin oxide TFTs can reach mobilities over 50 cm^2^/Vs but demonstrate anticlockwise hysteresis, making them inadequate for circuits [4]. Let us note that if anticlockwise hysteresis is inadequate for conventional oscillating circuits, AOSs such as nanoparticles of SnO_2_ may be useful for new applications such as synaptic mimicking devices for memory applications [27]. Other AOSs such as IZO have also been used for detecting human body-related ions under synaptic signals [28]. The goal of this manuscript is to evaluate the benefits of using another method than UV treatment to reach stable and high performance TFTs.

In the present manuscript, we report the use of NF_3_ plasma treatment on the surface of a solution-processed HfO_2_ gate insulator before the spin-casting of a-SnO_x_. Here, no UV treatment was used, in order to not mix the two effects. Reaching mobilities of ~35 cm^2^/Vs, we investigated various conditions to understand the impact of the treatment on the thin-film properties, such as roughness, crystallinity, and oxygen bondings, but also the performances of the TFTs. Plasma treatment is conventional in TFTs and can lead to performance enhancements. Kang et al. demonstrated that Ar/H_2_ plasma treatment on IGZO TFTs can enhance mobilities and photosensitivity [29]. Ar/H_2_ plasma treatment on IGZO TFTs can enhance mobility up to 26.5 cm^2^/Vs and also stability over bias stresses [30]. NF_3_ is usually used in the processes of cleaning [31], dry etching [32], or implementing F atoms and increasing conductivity, for example, in the source/drain region of coplanar TFTs [33]. Recently, combined NH_3_ and NF_3_ plasma treatments on ZnO surfaces demonstrated enhancements in both the mobility and stability of TFTs [23]. Even Ar/O_2_ treatment on a zirconium aluminum oxide gate insulator led to high performance TFTs [34]. Also, IGZO TFTs were fabricated by using N_2_O plasma treatment, and the high performances were assigned to the reductions in the defect density in the channel [35]. Let us note that no NF_3_ plasma treatment on the gate insulator of SnO_x_ TFT has been reported so far. 

To test the merit of the NF_3_ plasma-treated TFTs, we performed high-current stress (HCS) tests of 100 μA for 1 h. HCS instability has been mainly attributed to Joule heating in the device [36,37], but other reasons, such as gate insulator (GI)/channel interface quality, have also been reported [38,39,40]. The thermal conductivity difference between the channel and the other materials, especially the glass substrate, led to a local increase in the TFT channel region temperature to over 200 °C in poly-Si TFTs [37] and 240 °C in IGZO TFTs [41]. HCS leads then to high V_th_ shifts, SS degradation, and even to hump effects in TFT IV characteristics [36,37]. Active channel splitting and U-shaped TFTs have been proposed to effectively dissipate the heat and reduce Joule heating [36,41,42,43].

## 2. Materials and Methods

### 2.1. TFT Fabrication and Characterization

We fabricated bottom-gate top-contact TFTs, starting with 40 nm of Mo deposited by sputtering. Then, we spin-coated a 0.2 M solution made of HfCl_4_ mixed into ethylene glycol and acetonitrile (65, and 35% in vol.) to make the 95 nm of the HfO_2_ gate insulator. The spin-coating for HfO_2_ was performed at 2000 rpm, followed by a curing step at 100 and 240 °C. Each curing step was performed for 5 min. The temperature used helped remove the solvents: the boiling points of ethylene glycol and acetonitrile are ~197 and ~82 °C, respectively. The coating was repeated 4 times. Then, the sample was annealed at 350 °C for 2 h. At that point, we performed an NF_3_ plasma treatment for 0, 10, or 30 s on the HfO_2_ surface in a vacuum chamber. We input the NF_3_ gas at 30 sccm at 30 W, at a working pressure of 70 mTorr. The NF_3_/O_2_ ratio was 1:2. Then, we spin-coated a 0.16 M precursor solution made by mixing SnCl_2_ into ethylene glycol and acetonitrile (65 and 35% in vol.) at 4000 rpm to obtain a 9 nm a-SnO_x_ layer. All solution precursors and solvents were purchased from Sigma-Aldrich (Yongin-Si, Republic of Korea).

We then cured at 100 °C and at 300 °C. Each curing step lasted 5 min. After patterning and annealing at 300 °C for 2 h, we performed the via holes to reach the gate contacts. Finally, we sputtered and patterned 200 nm of IZO as the source/drain. We ended the fabrication through a post-annealing at 300 °C during 3 h. All patterning steps were performed by common photolithography. We measured the TFT IV and CV curves with a 4156C semiconductor parameter analyzer (Agilent Technologies, Inc. Santa Clara, CA, USA) and an Agilent E4980A precision LCR meter (Agilent Technologies, Inc., Santa Clara, CA, USA); respectively. We measured the gate insulator capacitance on a MIM structure (Mo-HfO_2_-Mo) with the aforementioned measurement system. The subthreshold slope was taken as the minimum value of ∂V_GS_/∂(log(I_DS_), and the V_th_ was taken as the value for which the current reached W/L × 10^−10^ A, where W and L were the width and length of the TFT. Also, the linear mobility μ_lin_ was extracted from the transfer curve with μ_lin_ = ∂I_DS_/∂V_GS_ × L/(W × C_ox_× V_DS_), where C_ox_ was the gate capacitance [44]. The TFT W and L were 50 and 10 μm, respectively. In total, 20 TFTs were evaluated for each condition. 

### 2.2. Thin Film Characterization

We measured the surface roughness by Atomic Force Measurement (AFM) with a tapping method on a 1 × 1 μm^2^ sample on a Xe-7 made by Park systems. We analyzed the O1s, F1s, and N1s spectra of a-SnO_x_ by X-ray spectroscopy (XPS) with a Phi 5000 Versaprobe by Ulvac-Phi (Ulvac-PHI, Kanagawa, Japan). The source was a monochromated Al Ka (at 1486.6 eV), and the beam spot was 100 × 100 μm^2^, with a detection limit of 0.5%. The measurement was performed at the HfO_2_/a-SnO_x_ interface. The crystallinity properties of the layers were measured by X-ray diffraction (XRD) (Rigaku, Tokyo, Japan) and grazing incident X-ray diffraction (GIXRD) (Rigaku, Tokyo, Japan), with a wavelength of 1.5405 Å. We note that all samples were made in the same manner as for the TFTs, except for the XRD measurement, where a 40 nm a-SnO_x_ layer was prepared. The thicknesses were measured with a profilometer.

## 3. Results

### 3.1. Device Characteristics

**Figure 1:**
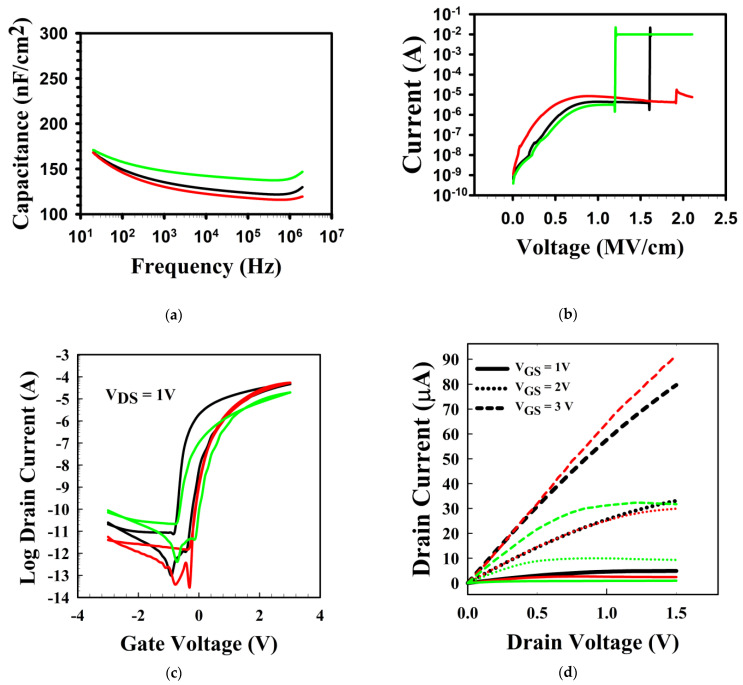
The electrical characteristics of NF_3_ plasma-treated HfO_2_ and of the TFTs. (**a**) The C-f characteristics of HfO_2_ over the range of 20–2 MHz, (**b**) the breakdown voltage of HfO_2_, (**c**) the transfer curves, and (**d**) the output curves. In all graphs, the black, red, and green curve relate to the 0 s, 10 s, and 30 s NF_3_ plasma-treated TFTs. In (**d**), each V_GS_ is shown by a different dashed line type.

Figure 1 shows the electrical characteristics of the HfO_2_ dielectrics and of the TFTs. Figure 1a,b shows the C-f characteristics and the breakdown voltages of the HfO_2_ dielectric with the various NF_3_ plasma treatment times of 0, 10, and 30 s. Figure 1a shows that the C-f does not significantly change with the treatment time, and, at 100 Hz, the capacitance value was at averages of 148, 147, and 159 nF/cm^2^ for the 0, 10 s, and 30 s NF_3_ plasma-treated HfO_2_ layers. Figure 1b shows that, with the treatment time increasing from 0 and 10 s to 30 s, the breakdown voltage is modified from 1.6 to 2.1 and to 1.2 MV/cm, respectively. Figure 1c shows the transfer characteristics of the TFTs with the various NF_3_ plasma treatment times of 0, 10 s, and 30 s. At V_DS_ = 1 V, both the non-treated and the 30 s-treated TFTs demonstrated an anticlockwise hysteresis of ~0.5 V, whereas the 10 s-treated TFT showed no hysteresis. Also, on average, the V_th_ shifts from −0.1 ± 0.1 to 0.1 ± 0.1 and to 0.2 ± 0.1 V for the 0 s-, 10 s-, and 30 s-treated TFTs, respectively. The SS values were 116 ± 14, 108 ± 13, and 118 ± 15 mV/dec. for the 0, 10 s, and 30 s-treated TFTs. The linear mobility was 31.0 ± 10.6, 35.0 ± 7.1, and 15.8 ± 4.5 cm^2^/Vs for the 0 s-, 10 s-, and 30 s-treated TFTs, respectively. Figure 1d shows the output curves of the TFTs. We can observe that they demonstrate clear saturation and that the 10 s-treated TFT demonstrated the highest current. The current measured at V_GS_ = 3 V, and V_DS_ = 1.5 V for the untreated, 10 s-, and 30 s-treated TFTs was 80, 91, and 32 μA, respectively. 

**Figure 2:**
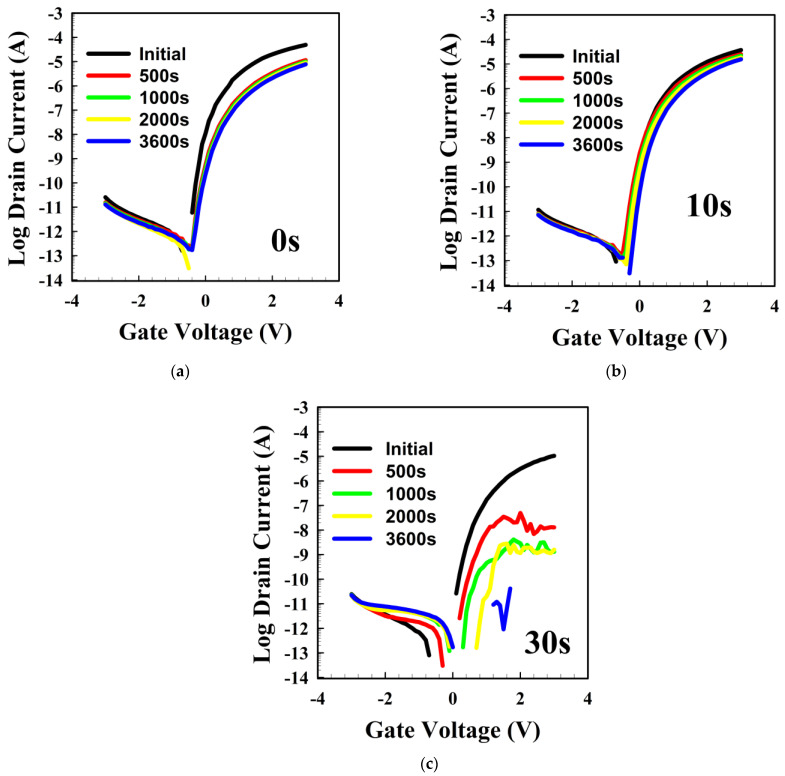
The stability under HCS of 100 μA of NF_3_ plasma-treated HfO_2_ surface in tin oxide TFT. (**a**–**c**) show the variations in the transfer curve of the TFT under HCS when the HfO_2_ layer was treated during 0, 10, and 30 s, respectively. The various measurement times and plasma treatment times are indicated within the figures. The transfer curves were measured at V_DS_ = 1 V.

We show the stability of the TFTs under the high current stress of I_DS_ = 100 μA in Figure 2. We observed a small V_th_ shift (ΔV_th_~0.4 V) and a decrease in the TFT ON-current during the stress. The untreated TFT demonstrated a 6.3-fold decrease in the ON-current, with an increase in SS, whereas the 10 s-treated TFT mostly showed a ~0.2 V V_th_ shift and a small decrease in the ON-current. The shift in the transfer curve is related to the trapping of electrons, whereas the decrease in the ON-current is associated with the creation of defects. On the other hand, the 30 s-treated TFT did not show a transistor effect after stress and even after a few days left in air (not shown here). The defects could be related to the weak -OM bonds, and, as the untreated TFT demonstrates an increase in SS, defects near/at the HfO_2_/a-SnO_x_ interface are also likely to occur. We now present the material properties. We will then discuss the reasons for the instabilities.

### 3.2. Thin Film Characteristics

**Figure 3:**
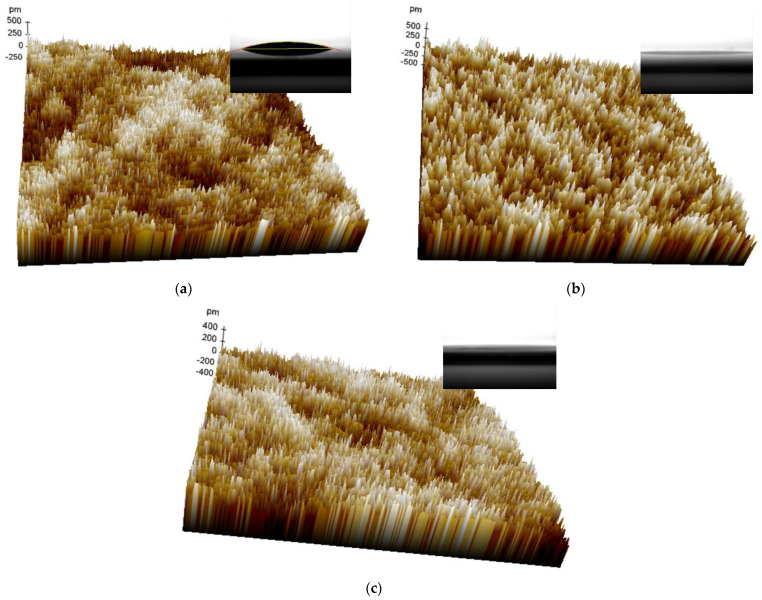
AFM images of NF_3_ plasma-treated HfO_2_ surface. (**a**–**c**) represent the surface after an NF_3_ plasma treatment of 0, 10, and 30 s, respectively. Insets: the water contact angle for each condition. The size of the surface is 1 × 1 μm^2^.

Figure 3a–c shows the AFM images of the 0 s, 10 s, and 30 s NF_3_ plasma-treated HfO_2_ surfaces. The RMS roughness values were 0.107, 0.159, and 0.108 nm, respectively, and the peak-to-valley roughness values were 1.007, 1.310, 0.903 nm, respectively. Therefore, the NF_3_ plasma treatment did not significantly modify the surface of the HfO_2_ and could not therefore explain the difference in TFT performances. The surface was smooth enough for electronics applications. Also, the water contact angle measured on the surface of the HfO_2_ surfaces (insets of Figure 3) revealed that the plasma treatment significantly reduced the contact angle from 17 for the untreated surface to ~0° for both treated surfaces. The treatment led to a better spread of the a-SnO_x_ solution [45]. The control of the surface’s wettability has also been shown to be a particular important factor in making high-performance organic TFTs [45,46]. Yet, there appears to be no significant difference in both treated TFTs, so the wettability cannot explain the variation in TFT performance.

**Figure 4:**
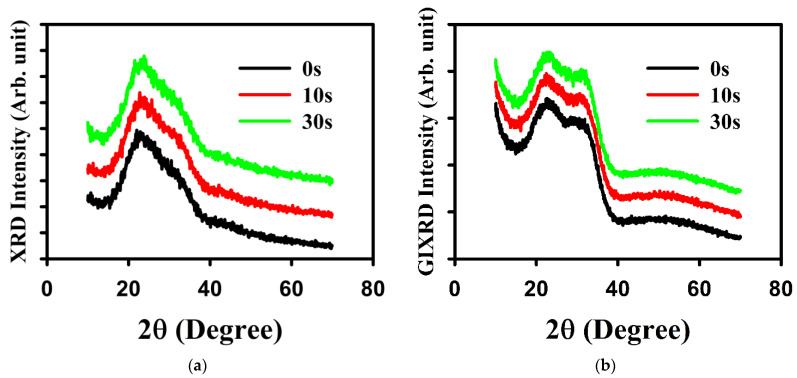
Crystallinity of the HfO_2_/a-SnO_x_ layers (**a**) measured by XRD and (**b**) measured by GIXRD. In (**a**), the tin oxide layer was 40 nm thick; in (**b**), the tin oxide layer was the same as in a TFT (9 nm). In both figures, the shoulder by 25° is due to the glass substrate, and the shoulder by 30° is due to HfO_2_.

Figure 4 shows the crystallinity of a-SnO_x_ on top of HfO_2_ with various treatment times. Figure 4a shows the XRD and demonstrates that the a-SnO_x_ layers are amorphous for all treatment times. Figure 4b shows the GIXRD measurements and that the tin oxide is amorphous in all treatment cases.

**Figure 5:**
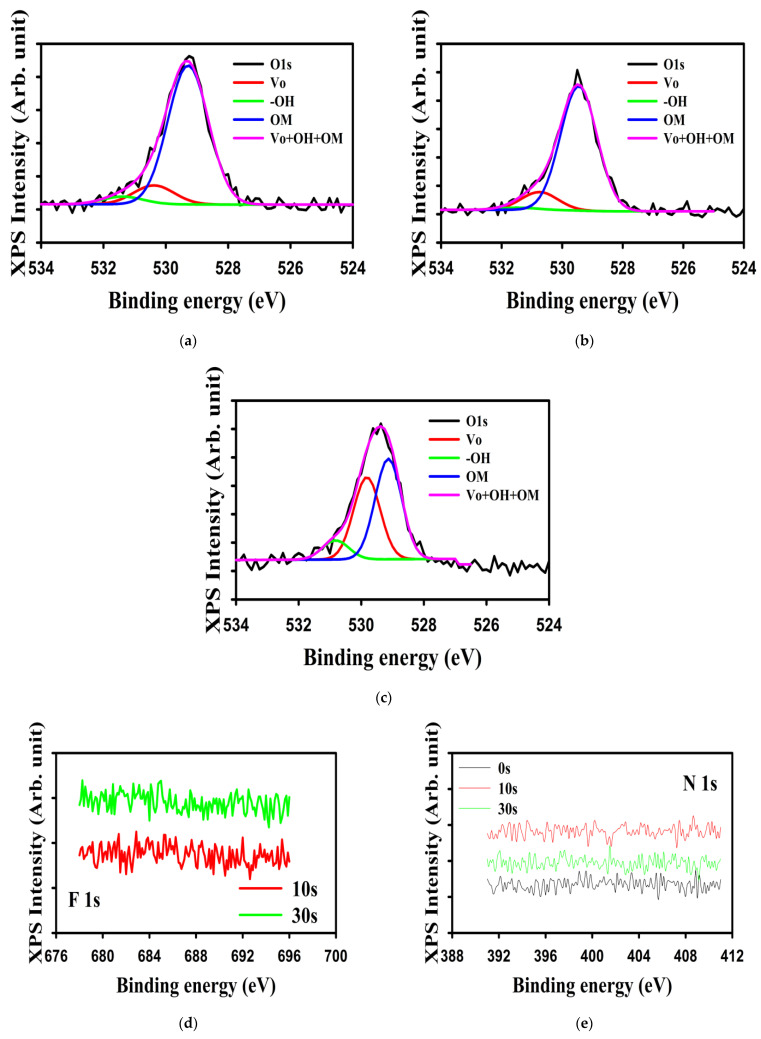
The O1s, F1s, and N1s spectra of tin oxide, as measured by XPS. The deconvolution of the O1s spectrum of (**a**) 0 s-, (**b**) 10 s-, and (**c**) 30 s-treated HfO_2_ layer shows the oxygen vacancy (Vo), -OH groups, and metal–oxygen (OM) bonds’ proportions. In each spectrum, we show the measured spectrum in black, the deconvoluted oxygen vacancy, the -OH groups, the OM bonds, and their sums in red, green, blue, and purple, respectively. (**d**,**e**) show the F1s and N1s spectra, respectively. In (**d**,**e**), the treatment times are indicated.

To understand the possible modifications in tin oxide, we investigated the bulk properties of tin oxide by first analyzing the O1s spectrum by XPS. Figure 5a–c shows the deconvolution of the O1s peak for the 0 s, 10 s, and 30 s plasma-treated HfO_2_ layers. The O1s peak can be deconvoluted into three main peaks: the metal–oxygen (OM bonds), the oxygen vacancy-related peak, and the -OH bonds [4]. The OM densities were 84.0, 86.2, and 49.8% and were located at 529.3, 529.5, and 529.1 eV for the 0 s, 10 s, and 30 s plasma-treated HfO_2_ layers, respectively. The Vo densities were 11.4, 12.4, and 40.7% and were located at 530.4, 530.8, and 529.8 eV for the 0 s, 10 s, and 30 s plasma-treated HfO_2_ layers, respectively. The -OH group densities were 4.6, 1.4, and 9.5% and were located at 531.4, 531.5, and 530.8 eV for the 0 s, 10 s, and 30 s plasma-treated HfO_2_ layers, respectively. Therefore, the 10 s NF_3_ plasma treatment helped to decrease the number of defects like the -OH groups and increase the number of OM bonds, whereas a too-long treatment increased the number of Vo defects, and the -OH groups, leading to a deteriorated tin oxide layer. The higher density of -OM will lead to an increased overlap of metal orbitals, increasing the mobility of the semiconductor, and the oxygen vacancies also provide carriers for the tin oxide layer [1]. We show the F1s and N1s spectra in Figure 5d and e, respectively. No F nor N were detected, suggesting that the atoms were not present in the a-SnO_x_ layer or below the detection limit. We note that in a previous report where NF_3_ and NH_3_ plasma treatments were used on ZnO, no F and no N were detected when only one treatment was used, but F was detected when both treatments were used [23]. 

Finally, we investigated the density of states by a method developed by Migliorato et al. [38,47]. To put it simply, the method relies on extracting the DOS by the following equation [47,48]

(1) $${N(E)}_{}=\frac{C_{G}-\frac{{dI}_{DS}}{{dV}_{GS}}\frac{L}{V_{DS}W{}_{lin}}}{q(1-\frac{C_{G}}{C_{ox}})d}$$

with

C_G_, q, and d being the gate capacitance per unit area at V_DS_ = 0 V, the charge of the electron, and the tin oxide thickness, respectively.

**Figure 6:**
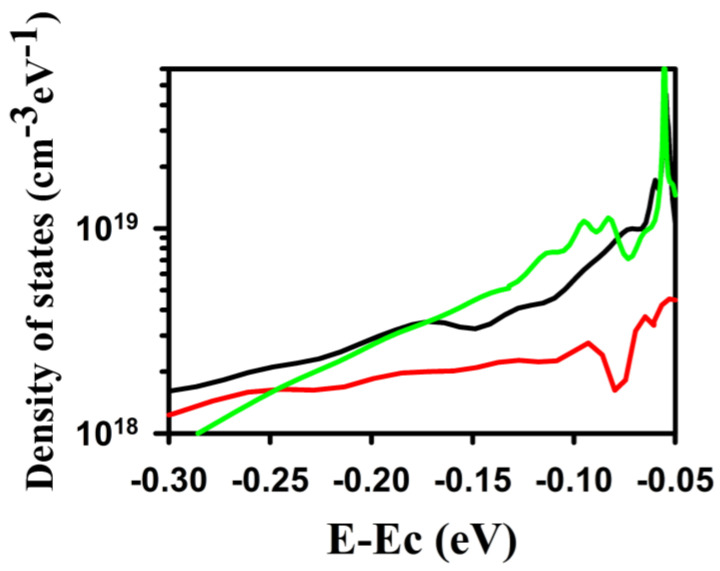
The extracted DOS of tin oxide. The black, red, and green lines represent the DOS for the 0 s-, 10 s-, and 30 s-treated TFTs.

For IGZO TFTs, the method is valid for a range of energy from Ec down to at least Ec-0.3 eV [47]. Figure 6 shows the result of such an extraction. The DOS is in the 10^18^–10^19^ cm^−3^ eV^−1^ range, similar to the values reported by the TCAD simulation [49]. The 10 s-treated sample demonstrates a lower DOS than the untreated sample, and the 30 s-treated TFT is down to Ec-0.25 eV, whereas the 30 s shows the highest DOS from Ec-0.07 to Ec-0.17 eV. So, a 10 s NF_3_ treatment will positively impact the DOS inside the bandgap of the tin oxide material, whereas a long plasma treatment will affect negatively the DOS near the conduction band. These defects will negatively impact the TFTs by pinning the Fermi level and not allowing high mobility in the TFTs. So, considering that the 30 s NF_3_ plasma-treated HfO_2_ has a smaller breakdown voltage than other conditions, and the fact that the 30 s plasma-treated TFT has a higher DOS close to the Ec compared to the untreated TFT, could further explain the poor TFT characteristics.

## 4. Discussion

The main focus of this paper has been how NF3 plasma treatment on the gate insulator affects the channel region on top of it. Let us first note that AOSs are subject to a mobility–stability tradeoff [50], and the high mobility AOSs are more sensitive to process conditions. We now discuss the various aspects, starting with the effect of the plasma treatment. NF_3_ plasma is usually performed in the source/drain region in order to increase the conductivity. For example, Um et al. demonstrated a decrease in resistivity from 10^2^ to 3 × 10^−3^ Ohm·cm, leading to a further increase in Hall mobility from 8 to 22 cm^2^/Vs [33]. Also, treatments on the gate insulator usually decrease defects at the GI/channel interface and/or decrease the contact angle by improving the wettability before the deposition of the channel on top of it [45,46]. The Ar/O_2_ in zirconium aluminum oxide allowed for the decrease in defect states in the insulator, and an improvement in the material allowed for a more stable TFT [34]. As introduced before, plasma treatments can be also effective on the channel to decrease defect density within the channel. CF_4_ was, for example, used on top of the IGZO channel region, with a mild power of 15 W, to ensure no damage to the layer [51]. CF_4_ has also been recently used for IGZO TFTs and demonstrated improvements in all kinds of gate bias stresses and illumination stresses [52]. Regarding our experiment, we describe the impact not only on the gate insulator but also on the DOS of the channel material. The AFM results demonstrated that the RMS roughness remains below 1 nm for all conditions, but the peak-to-valley was actually higher for the 10 s treatment. So, the benefit of the treatment is therefore as shown by the XPS analysis by the increase in the density of -OM bonds at the interface.

Among the various methods to evaluate the DOS in oxide semiconductors, the one we chose was a rapid and reliable method. As introduced above, the method allowed for a precise extraction below the Ec and could also give information within the bandgap down to Ec-1 eV [47,48]. Due to the reduced range of the TFT measurements, our scope was reduced to the region near the Ec. We note that we observed high DOS in our material in a previous study and donor states near the E_C_ [49]. The donors are related to the presence of oxygen vacancies, which, by ionizing, leads to an increase in electrons in the channel [1,49]. So, to have a better understanding of the DOS in our TFT, the next step would be to undergo experiments with SiO_2_ (for example) as the GI to increase the range available to obtain information about the DOS. Another method would be to use a multiple frequency measurement method, which has been shown to adequately describe the DOS [53]. Finally, let us note that materials and structures also impact TFT performances. A next approach is to evaluate the source/drain electrodes on the TFT performances, even though materials seem to have no to small effects on the performances, and they may influence the stability by affecting the potential creation of defect states near the drain regions [54,55,56].

Let us now discuss the stability of our TFTs. As introduced above, Joule heating would greatly affect the behavior of the device under HCS [57]. So, the ability to dissipate the heat (as it could indicate the thermal conductivity) is an important factor for the stability of the device. SnO_2_, in a rutile structure, has a thermal conductivity depending on the axis: 98 W/(Km) along the c-axis and 55 W/(Km) perpendicular to the c-axis [58,59]. Also, polycrystalline SnO_2_ demonstrated a thermal conductivity between 25 and 40 W/(Km) [60]. As a comparison, IGZO demonstrates a thermal conductivity of ~1–8 W/(Km) in both an amorphous and crystalline state (i.e., CAAC-IGZO) [61,62,63]. The porosity and also the fabrication process of the material are important parameters in the thermal conductivity values [63]. The solution-processed IGZO would lead to similar values at room temperature as sputtered films, but their behaviors at lower temperatures would differ. We previously demonstrated that our a-SnO_x_ had a smaller density of 5.29 g/cm^3^ than the theoretical value of 6.95 g/cm^3^ [4]. Therefore, assuming a lower thermal conductivity than the reported above value, but still higher than IGZO value, we believe that the Joule heating may have a role but have a lower effect than in IGZO TFTs. Indeed, we previously reported the effect of positive bias stress on the a-SnO_x_ TFTs, and they demonstrated a similar decrease in the ON-current as the untreated TFTs [4]. In a-IGZO TFTs, V_Th_ shifts and higher SS values were observed under HCS compared to PBS [36]. The same phenomenon was observed in poly-Si TFTs, which showed an even higher thermal conductivity of 168 W/(Km) [37]. Therefore, we could rule out the Joule heating impact in our TFTs. Let us note that, at room temperature, the channel thickness plays a role in the stability of the TFT under high current stress, and the thicker the more stable due to a higher probability of the recombination of the drain region’s impact-generated holes and electrons in IGZO TFTs [64], but IZTO TFTs are rather stable [65]. Under simple PBS and negative bias illumination stress, IGTO TFTs demonstrate high performances at an optimized thickness of 15 nm [10].

Degradation of the TFTs has also been understood by the quality of the gate insulator (GI)/channel region quality [38,39,40,66]. For example, under HCS, charge trapping near/at the GI/IGZO channel of IGZO TFTs was demonstrated to be inherently governed by the interface quality. The higher number of defects lead to the higher degradation of the TFTs [39]. Also, Kim et al. showed that, in IGO TFTs, the diffusion of Al atoms from the Al_2_O_3_ GI into the IGO channel region could lead to an improvement in TFT characteristics [40]. Aluminum introduced in IZO also demonstrated the control of the TFT IV characteristics by controlling the flat-band voltage [67].

Therefore, we conclude that the degradation phenomenon observed is, in part, due to the HfO_2_/a-SnO_x_ interface defects. The defects present in our TFT GI/a-SnOx interface are -OH groups (electron trapping), weak -OM groups that could be easily broken, and oxygen vacancies [49]. The -OH groups are responsible for V_th_ shift, and the weak -OM groups and Vo are responsible for the degradation of the TFT ON-current during the HCS. The analysis of the O1s of a-SnO_x_ near the HfO_2_/a-SnO_x_ reveals that, compared to the untreated TFT, Vo and -OH density increase in the 30 s NF_3_ plasma-treated TFT, -OH groups decrease, and -OM density increases in the 10 s-treated TFTs. Let us not forget that the 10 s-treated TFT also demonstrates a lower DOS than the other TFTs, also explaining, in part, the improved TFT performances.

Therefore, the NF_3_ plasma treatment has a surface effect at the GI/a-SnO_x_ interface but also a bulk effect in a-SnO_x_ by effecting the DOS. The NF_3_ plasma treatment affects the tin oxide significantly. During the treatment, O_2_ and NF_3_ may interact to form F radicals and NO molecules [68]. We suggest that the dissociated NF_3_ molecules from the plasma are mixed within the tin oxide precursor solution during the spin-coating, modifying the tin oxide layer, and may diffuse out during the curing and annealing steps. Therefore, the improved TFT performances of the 10 s-treated TFTs can be explained by an increase in the MO bonds, a decrease in -OH bonds, and a decrease in the DOS compared to an untreated sample. A 30 s long treatment increases the Vo defects and -OH groups, decreases the OM bond density, and increases the DOS compared to an untreated HfO_2_ layer, leading to the deterioration in TFT performances.

## 5. Conclusions

We studied the impact of NF_3_ plasma treatment on the HfO_2_ gate insulator before the spin-coating of the tin oxide channel layer. Contrary to expectations, the treatment only slightly affected the HfO_2_ layer and dramatically impacted the tin oxide layer. Indeed, for HfO_2_, the RMS roughness and capacitance values were similar with the various treatment times, whereas the breakdown voltage was slightly impacted with the treatment times. Compared to an untreated TFT, a 10 s-treated TFT showed a decrease in DOS and a similar mobility of ~35 cm^2^/Vs. The 30 s-treated TFT showed a significant decrease in the mobility (~15 cm^2^/Vs) and a higher DOS than the untreated TFT. Also, the V_th_ was impacted for the 30 s-treated TFT, due to a lower number of electrons available in the channel, due to a higher number of defects in the tin oxide channel. Also, the 10 s-treated TFTs showed a hysteresis of ~0 V, whereas the untreated and the 30 s-treated TFT showed a hysteresis (~0.5 V). The 10 s-treated TFT also demonstrated a higher stability under HCS than the untreated TFT, whereas the 30 s TFTs showed no currents after HCS. We rejected Joule heating as the main reason and attributed the HfO_2_/a-SnO_x_ interface quality as the main reason. Due to the absence of N and F in the a-SnO_x_ layer, we suggested that the NF_3_ molecules affected the density of states, and the oxygen environment in the a-SnO_x_ layer, without changing the crystallinity, and may diffuse out during the curing and annealing steps. The present study represents an alternative to the previously used UV processing HfO_2_. Even though UV treatment leads to higher mobility, the high hysteresis of the TFTs could not lead to circuit manufacture. The next step is to include both UV and NF_3_ plasma treatments to obtain high performances and stable TFTs and circuits. Other routes to improve the a-SnO_x_ TFTs are the use of other contact materials, passivation layers, and TFT structures.

## Data Availability

Data are contained within the article.
